# The bacterial species’ degradation activities at maximum threshold doses of glyphosate across different pH levels and temperature glyphosate biodegradation by soil bacteria at high doses under variable pH and temperature

**DOI:** 10.3389/fmicb.2025.1668968

**Published:** 2025-12-04

**Authors:** Tinatin Doolotkeldieva, Saykal Bobusheva, Mahabat Konurbaeva

**Affiliations:** 1 Plant Protection Centre, Kyrgyz National Agrarian University, Bishkek, Kyrgyzstan; 2 Plant Protection Department, Kyrgyz Turkish Manas University, Bishkek, Kyrgyzstan

**Keywords:** glyphosate-contaminated soil, threshold concentrations of the herbicide, degradingbacteria, the bacteria’s biodegradation ability, bacterial activity at high concentrations

## Abstract

Glyphosate (N- (phosphonomethyl) glycine) is an organophosphorus compound and one of the most widely used herbicides worldwide. However, concerns about its environmental impact have prompted researchers to investigate its degradation process. This study addresses several key questions, particularly concerning the concentrations of herbicides that can inhibit the activity and disrupt the functions of degrading bacteria. Specifically, the research aims to identify the maximum glyphosate concentrations that hinder the growth and development of degradative bacteria. Three bacterial strains were tested: *Stenotrophomonas* sp. Ps-B, *Lysinibacillus fusiformis* SA-4, and *Enterobacter cloacae* SB-2. Glyphosate degradation was evaluated at concentrations of 50, 100, 200, 1,000, 2,500, 5,000, and 10,000 mg/L across liquid, solid, and soil media, with different pH levels and a constant temperature of 28° C. Controlled samples—both without added bacteria and containing only glyphosate—ensured the reliability of the results. Bacterial cell density was measured after 24 and 72 h using a spectrophotometer, and microbiological culture techniques were employed to quantify colony- forming units. The minimum inhibitory dose of glyphosate affecting the viability of degrading bacteria on solid nutrient media was determined via the diffusion agar method. For each strain, a specific minimum inhibitory concentration was established: 10000 mg/L for *Lysinibacillus fusiformis* SA-4, 5,000 mg/L *for Stenotrophomonas* sp. Ps-B, and 10,000 mg/L for *Enterobacter cloacae* SB-2. *Stenotrophomonas* sp. Ps-B has shown superior activity at pH levels of 8.0, 9.0, and 10.0–an important consideration for the biodegradation of alkaline soils. Complete degradation of high herbicide doses required an additional incubation period of at least 40 days. Overall, the study demonstrates that these bacterial isolates are effective in remediating soil after glyphosate application, helping prevent herbicide accumulation. Consequently, they hold promise for bioremediation of glyphosate-contaminated soils, even at high concentrations and over extended periods.

## 1 Introduction

The variety of herbicides available for targeting different types of annual or perennial weeds continues to grow yearly. Unfortunately, an expanding list of plants has developed resistance to these chemicals (Dill et al., 2010). Glyphosate (N-phosphomethylglycine) is an organophosphorus compound that stands out among herbicides due to its exceptional effectiveness in controlling various weeds. This efficacy has made it one of the most widely used herbicides in the world (Duke and Powles, 2008; Benbrook, 2016; Botten et al., 2021; Cuhra et al., 2016). Glyphosate is absorbed through the leaves rather than the roots and acts both by contact and systemically, moving from the leaves to the roots within the plant. Rapid results occur in areas where there is active cell growth and division (Zobiole et al., 2011; De María et al., 2006). Glyphosate, first discovered in Switzerland in 1950, was initially used as a chelator. Its primary function was to bind and remove calcium, magnesium, manganese, copper, and zinc from solutions; this use has evolved significantly over the years (Casida, 2017; Swarthout et al., 2018). Monsanto began commercializing it in 1974 for its broad-spectrum properties in different herbicide formulations (Myers et al., 2016). The mechanism of herbicidal activity of glyphosate is based on the inactivation of the key enzyme 5-enolpyruvylshikimate-3-phosphate synthase (EPSPS) in plants, disrupting the biosynthesis of aromatic amino acids (phenylalanine, tyrosine and tryptophan) and the subsequent production of proteins, thereby killing the plant within 1–3 weeks (Gill et al., 2018; Kanissery et al., 2019; Souza et al., 2025).

The use of glyphosate has increased significantly. In 1994, approximately 16 million kilograms of glyphosate were applied, but by 2014, this figure had surged to 79 million kilograms, primarily due to the development of weed resistance (Benbrook, 2016). As a result, farmers employ higher frequencies and doses of herbicides throughout the growing season to manage resistant weed species. Globally, glyphosate use in 2025 is projected to reach between 740,000 and 920,000 tons (Matozzo et al., 2020). Glyphosate-based herbicides are typically applied at high concentrations, ranging from 6.7 to 8.9 kg per hectare. In conventional agriculture, the application rate is usually between 0.53 and 1.0 kg per hectare (Villamar-Ayala et al., 2019). This practice results in the accumulation of chemical residues in the soil, harming soil ecosystems and contributing to severe environmental pollution (Jesús et al., 2023). This herbicide can persist in the soil for extended periods. Its residual quantities may accumulate in water sources, non-target plants such as food, and in the bodies of mammals, birds, and humans through food chains, inhalation, and skin contact (Gill et al., 2018; Defarge et al., 2018; Sesin et al., 2021; Krüger et al., 2014; Torretta et al., 2018). Glyphosate and AMP residues have been reported in the urine of the USA (in 60–80% of samples) and Europe (in 44% of samples), with average concentrations of 2–3 g/L and less than 1 g/L, respectively (Soukup et al., 2020; Niemann et al., 2015).

Research suggests that exposure to glyphosate is linked to various health issues, including cancer cell lymphomas (Leon et al., 2019), neurological disorders (Hoppin et al., 2017), and an increased risk of mortality from Parkinson’s disease in humans (Caballero et al., 2018). Additionally, prenatal exposure to glyphosate has been associated with pregnancy loss (Von Ehrenstein et al., 2019). Studies have also demonstrated the toxic effects of glyphosate and its metabolites on soil microbial communities, which reduces nutrient availability in the soil (Gill et al., 2017; Koskinen et al., 2016).

The evidence suggests that glyphosate harms weed biodiversity and poses risks to non-target organisms and human health. This situation highlights the urgent need to address the consequences of glyphosate use. Therefore, it is essential to find environmentally safe methods for removing its residues and metabolites from the environment.

Among the various methods available, the biodegradation of glyphosate using microorganisms is considered safe, cost-effective, and reliable for removing this xenobiotic from water and utilizing glyphosate as essential nutrients, including nitrogen, carbon, and phosphorus. Recent studies have highlighted the potential of certain bacterial isolates to promote the biodegradation of glyphosate. Evidence suggests that some bacterial strains possess specific enzymatic pathways that can break down this herbicide into less harmful by-products.

Some of the most active and well-known glyphosate-degrading bacterial species include *Achromobacter* spp., *Agrobacterium radiobacter, Alcaligenes* sp. *GL*, *Arthrobacter* spp., *Bacillus cereus* CB4, *Ochrobactrum* spp., and *Pseudomonas* spp. Additionally, notable fungal species that degrade glyphosate are *Aspergillus niger*, *Aspergillus oryzae* A-F02, *Penicillium chrysogenum*, and *Trichoderma harzianum* (Rossi et al., 2021; Fu et al., 2017; Wijekoon and Yapa, 2018; Souza et al., 2025).

Degrading bacteria possess a unique enzyme called glyphosate oxidoreductase (GOX), which breaks down the glyphosate molecule into glyoxylate. This process results in the complete oxidation of glyphosate, producing carbon dioxide. An intermediate metabolite known as aminomethylphosphonic acid (AMPA) is also formed. AMPA is further hydrolyzed into phosphate and methylamine by the enzyme carbon-phosphorus lyase (C-P-lyase) (Souza et al., 2025).

Depending on the bacterial species, two primary metabolic pathways are responsible for glyphosate degradation. In one pathway, certain bacteria utilize glyphosate oxidoreductase to oxidize glyphosate, resulting in the formation of intermediates and end products such as aminomethylphosphonic acid (AMPA) and formaldehyde (Zhan et al., 2018). Alternatively, other bacteria employ the C-P lyase pathway, where the enzyme carbon-phosphorus (C-P) lyase cleaves the carbon-phosphorus bond, producing sarcosine (a simple amino acid derivative) and inorganic phosphate (Aslam et al., 2024). The genes encoding these enzymes differ among bacterial genera, highlighting the diversity of biodegradation mechanisms (Singh and Christina, 2022).

The genetic regulation of enzyme efficiency in various bacterial species shapes how microorganisms adapt to diverse environmental conditions. *Bacillus* species exhibit optimal degradation at neutral pH (Yu et al., 2015), whereas tolerance and degradation of glyphosate by *Stenotrophomonas, Lysinibacillus, Enterobacter* under stress conditions demonstrates resilience in more extreme environments with higher pollutant concentrations (Ibrahim et al., 2023). These differences highlight the advantage of using mixed-species bioremediation strategies. By combining species with complementary resistance and adaptive traits, degradation can be optimized to suit specific polluted environments.

Several studies have examined the capacity of different bacterial species to degrade glyphosate into intermediate and final metabolites. For instance, *Stenotrophomonas acidaminiphila* strain Y4B demonstrated efficient glyphosate degradation at concentrations ranging from 50 to 800 mg/L, achieving over 98% removal within 72 h at 50 mg/L. This strain utilized the AMPA pathway, initially cleaving the C-N bond, followed by AMPA breakdown and subsequent metabolism. Under soil conditions, strain Y4B significantly accelerated glyphosate degradation, achieving removal rates of 71.93% in sterile soil and 89.81% in non-sterile soil at 400 mg/kg within 5 days (Li et al., 2022).

Other researchers have found that the bacterial strains *Serratia liquefaciens*, *Klebsiella* var*iicola*, *Enterobacter cloacae*, *Pseudomonas aeruginosa*, and *Enterobacter ludwigii* can effectively degrade 95–98 mg/kg of glyphosate in soil and 93–96 mg/kg in liquid media over 28 days of incubation, even under varying environmental conditions. Optimal glyphosate decomposition occurred under aerobic conditions at pH 7,0 and 40 °C, with 10% soil moisture and a sandy loam texture (Mohy-Ud-Din et al.2024).

Other researchers have reported compelling scientific and practical findings regarding glyphosate degradation. Experimental evidence shows that the *Bacillus cereus 6P* strain can release inorganic phosphate into the culture supernatant while simultaneously accumulating polyphosphate inside its cells during glyphosate biodegradation. Notably, this is the first report describing the biological conversion of glyphosate into polyphosphate. The authors highlight that the production of this biopolymer offers a significant ecological advantage, allowing the strain to adapt to environments exposed to glyphosate. The polyphosphate yield reached 4 mg/L, and the kinetic constant for glyphosate biodegradation was calculated at 0.003 h^−1^ s^−1^. Furthermore, the *B. cereus* 6P strain is considered a promising candidate for developing innovative biotechnological methods to produce polyphosphate through glyphosate biodegradation (Acosta-Cortés et al., 2019).

In recent years, glyphosate has been widely used for weed control across agricultural lands in our country, which are dominated by meadow-gray and high-clay-content gray soils. Developing effective bioremediation strategies requires targeted research to identify bacterial strains that can tolerate extreme conditions, such as alkaline environments and soils heavily contaminated with adsorbed herbicides. Research indicates that microbial biomass plays a crucial role in accelerating herbicide degradation, underscoring the significance of these microorganisms in sustainable agriculture. Gaining insight into the mechanisms by which bacteria degrade glyphosate is crucial for designing effective bioremediation strategies that minimize the environmental risks associated with glyphosate application.

This study investigates crucial issues, focusing on the herbicide concentrations that inhibit bacterial degradation and disrupt microbial function. It is important to determine the maximum threshold at which bacteria can metabolize herbicides as a nutrient source without experiencing toxicity or cell death. Additionally, it is important to assess how pH and temperature influence the degradative activity of bacteria exposed to high doses of glyphosate. This study seeks to determine the maximum glyphosate concentrations that inhibit the growth and activity of degradative bacteria under different pH and temperature conditions, as well as to identify bacterial species tolerant to extreme environments.

## 2 Method and materials

### 2.1 Degrading bacteria used in the experiments

Three bacterial strains, *Stenotrophomonas* sp. Ps-B, *Lysinibacillus fusiformis* SA-4, and *Enterobacter cloacae* SB-2, were selected for trials in media with varying herbicide concentrations. These bacterial species were isolated in the laboratory from heavily pesticide-polluted soil in southern Kyrgyzstan (Table 1) and identified to the species using classical microbiological and PCR analysis. The 16S ribosomal RNA (rRNA) gene sequences were deposited in the GenBank and DB of the National Center for Biotechnology Information nucleotide sequence databases (Doolotkeldieva et al., 2018).

**Table 1:** The origin of the bacterial isolates used in the bioaugmentation and bioremediation field tests (Doolotkeldieva et al., 2024).

Collection name and number	Strain isolation site	Bacterial strain with maximum homology based on partial 16S rRNA analysis	Identity (%)
SA-4	Suzak dumping zone soil	*Lysinibacillus fusiformis*	97.0
Ps-B	Suzak dumping zone soil	*Stenotrophomonas* sp.	99.0
SB-2	Suzak dumping zone soil	*Enterobacter cloacae*	94.0

They were sequentially screened *in vitro* and *in vivo* for their high viability and metabolic activity to degrade pesticides in contaminated soils. In the screening biotests, they demonstrated high viability and metabolic activity in soils with medium and high pesticide content (Doolotkeldieva et al., 2021; Doolotkeldieva et al., 2024).

### 2.2 Storage and activation of bacterial culture

In the first stage, bacterial cultures were revived from frozen storage at −40 °C in a laboratory collection. The cultures were re-seeded in sterile water and transferred to a liquid nutrient medium. They were grown with constant stirring (220 rpm) on a shaker for 24 h. After this period, the cultures’ homogeneity and the cells’ structure were examined using microscopy on prepared smears. To ensure the viability of each strain, a homogeneous and pure culture was cultivated in meat-peptone broth for 48 h on a shaker (220 rpm). Following microscopy to confirm complete viability, the strain was selected for incubation with the pesticide.

### 2.3 Herbicide used in the degradation experiments

Glyphosate is an organophosphorus compound used as an herbicide. It is widely used in many countries around the world, including those in Central Asia. This solid, non-volatile substance dissolves in water (12 g/L) and alcohol but has low solubility in organic solvents. Technical glyphosate is typically found as a white powder with a concentration of at least 95% (European Commission, 2023). We used high-purity, solid glyphosate (95% TC) herbicide for weed control, sourced from Metorri Chemical (Shanghai, China), in our experiment.

### 2.4 *In vitro* experiments in a liquid medium

The experiments were conducted in a liquid mineral medium composed of the following components: (NH_4_)_2_SO_4_ (1 g), K_2_HPO_4_ (0.8 g), KH_2_PO_4_ (0.2 g), MgSO_4_·7H_2_O (0.2 g), CaCl_2_·2H_2_O (0.1 g), (FeCl_3_)·6H_2_O (0.05 g), (NH_4_)6Mo7O_2_4·4H_2_O (0.01 g), and water (1,000 mL, pH 7.0). In this medium, microbial cultures were incubated with varying concentrations of glyphosate herbicide. Before the experiments, glyphosate solutions were prepared and dissolved in water, with concentrations ranging from 50 mg/L to 20,000 mg/L. Additionally, 5 mL of bacterial culture was introduced into flasks containing 100 mL of the mineral medium and incubated on a shaker (BIOSAN-80, United States). Each experiment was replicated three times to ensure accuracy under different conditions: pH levels of 7.2, 8.0, 9.0, and 10.0, temperatures of 28 °C, with a stirring speed set at 150 rpm. After 24 and 72 h, samples were collected and measured using a spectrophotometer. Microbiological culture techniques were also employed to determine the colony-forming units of the bacteria involved.

### 2.5 Determination of the minimum inhibitory concentration of glyphosate for bacteria in a solid medium

The Environmental Protection Agency issues regulations to establish, modify, suspend, or repeal tolerance limits (TLs)—the maximum allowable concentrations of pesticide residues in food. These limits are set at levels that ensure a reasonable certainty of no harm to consumers (EPA, 2025). This Agency establishes acceptable glyphosate residue limits for many crops consumed by humans and animals—including corn, soybeans, oilseeds, grains, fruits, and vegetables—with permissible levels ranging from 0.1 to 400 parts per million (ppm), (Environmental Topics, Chemicals, Pesticides and Toxics Topics) (European Food Safety Authority, 2023). The threshold concentration of glyphosate in soil is 0.5 mg/kg (FAO, 2004, 2011). Local farmers have confirmed that the maximum concentration of glyphosate used in agricultural fields ranges from 1,200 to 1,500 mg per hectare, depending on the soil and climatic conditions of the region. For the first series of tests in liquid media, solution concentrations of 50, 100, 200, 1,000, and 5,000 mg/L were selected, several times higher than the standard dose. Microorganism growth was abundant at solution concentrations of 50, 100, 200, and 1,000 mg/L, with noticeable inhibition occurring only at 5000 mg/L. For the second test series in solid medium, glyphosate concentrations were increased to 5,000 mg/L, 10000 mg/L, and 20,000 mg/L respectively, corresponding to 10,000, 20,000, and 40,000 times the standard dose of 0.5 mg/kg. Laboratory strains SB-2, SA-4, and Ps-B were inoculated onto Petri dishes. Once the agar solidified, four wells were created in each dish, and 0.3 mL of a solution containing glyphosate at concentrations of 5,000, 10,000, and 20,000 mg/L was added to each well. The Petri dishes were then incubated at 27 °C for 24 h to facilitate the diffusion of glyphosate. Three replicates of each strain were meticulously carried out for each concentration to ensure the reliability of the results. Distilled water was employed as a control.

### 2.6 Evaluating the ability of selected bacterial species to degrade glyphosate under soil conditions

#### 2.6.1 Characteristics of the soil type used in the experiment

The meadow-grass soils in the northern part of the Chuy Valley develop under conditions of prolonged ground moisture, which is influenced by ephemeral grass and sedge vegetation. These meadow-grass soils typically have a light gray color in both the humus horizon (A) and the illuvial horizon (B), often displaying a bluish tint. In the lower horizon (C), which is close to the parent rocks, rusty ochre specks can be observed.

The soil contains between 1.3 and 3.5% humus, 0.2 to 0.4% total nitrogen, 0.2 to 0.3% phosphorus, and 2 to 2.5% potassium. Its absorption capacity ranges from 12 to 15 mg-eq per 100 g of soil, with calcium being the predominant component of the absorbed bases. In contrast, gray-meadow soils are darker in color, with horizon B also showing a bluish tint along with additional spots.

**Figure 1:**
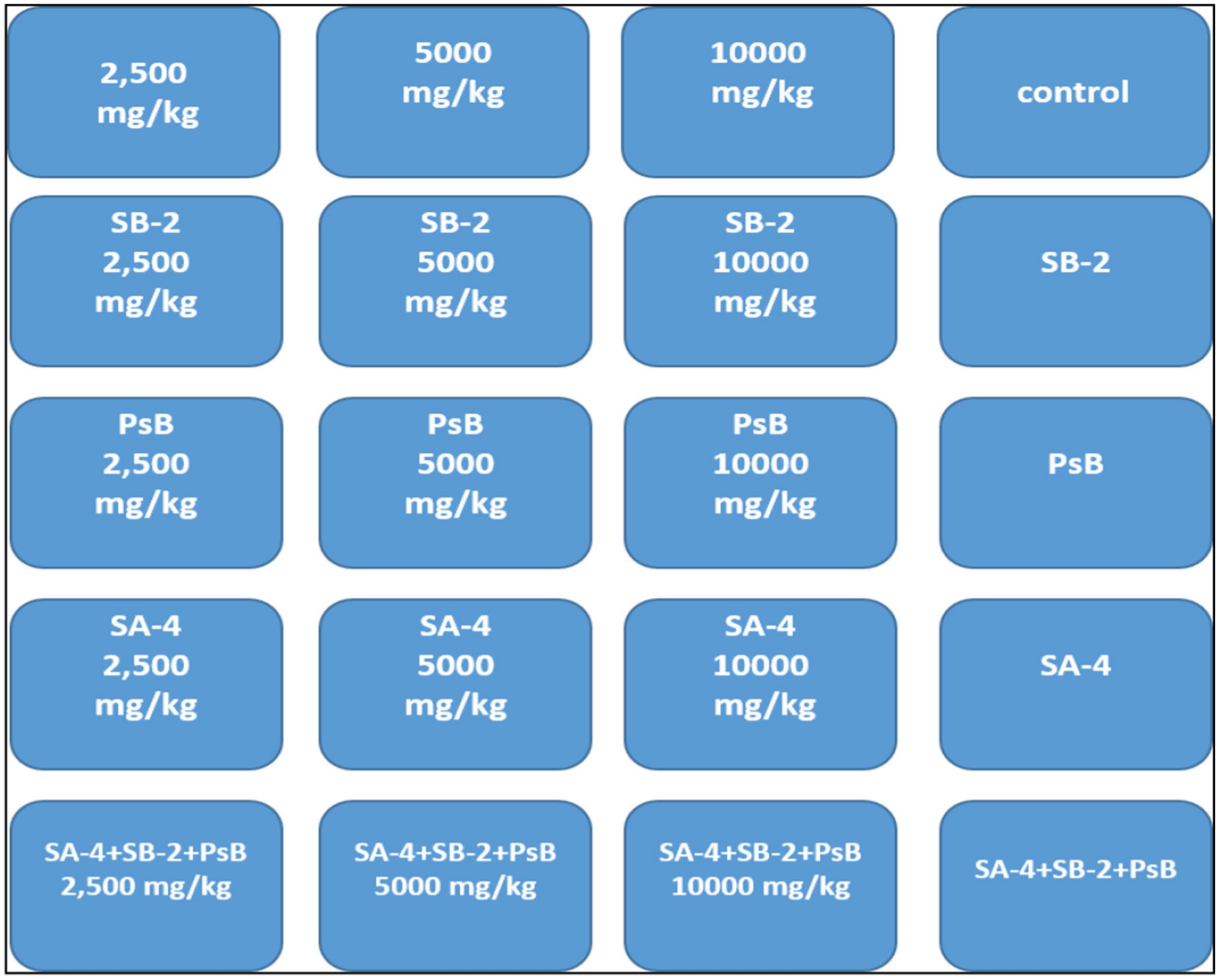
The experiment involves testing soil with different concentrations of glyphosate and single or combined bacteria.

After the initial screening, the ability of bacteria to degrade glyphosate was tested in a laboratory setting using sterile soil that had not previously been treated with the herbicide. The soil was disinfected by autoclaving it for three consecutive days at 121 °C for 20 min each day. Different concentrations of glyphosate were mixed into the soil by adding sterile water and a 10 mL bacterial suspension. Three isolates’ ability to degrade glyphosate at 2500 mg/kg, 5,000 mg/kg, and 10,000 mg/kg was evaluated in soil. Control variants included samples without added bacteria and those with only glyphosate (Figure 1). Soil samples were collected every 10 days and seeded onto nutrient media using a serial dilution method. After 40 days, wheat was planted in these soils to verify the study’s final results.

### 2.7 Identification of the final products of glyphosate decomposition in soil by the tested bacteria

After 40 days, the intermediate and final glyphosate products decomposed by the isolates in the soil were assessed. To conduct this assessment, the salt extraction method complies with GOST 26483 (Christmas, 2014). The determination of mobile phosphorus compounds is based on their extraction from the soil using an extractant solution. The extractant consisted of a potassium chloride solution with a concentration of 1.0 mol/L. The determination of mobile phosphorus compounds is based on their extraction from the soil using an extractant solution in a ratio that varies depending on soil and regional conditions, resulting in the formation of a blue phosphomolybdenum complex. Polyphosphates and esters do not participate in the reaction. The colored sample is measured visually for colorimetry using a color control scale. After extraction, the mobile phosphorus compounds are identified by forming a blue phosphomolybdenum complex:

$$\begin{array}{l} {{\mathrm{HPO}}_{4}}^{2-}+2{{\mathrm{NH}}_{4}}^{+}+12{{\mathrm{MoO}}_{4}}^{2-}+23H^{+}\\ ={\left({\mathrm{NH}}_{4}\right)}_{3}[{\mathrm{PMo}}_{12}O_{40}]+12H_{2}O\;\text{yellow} \end{array}$$

When subjected to a reducing agent—ascorbic acid in the presence of potassium antimony tartrate—the resulting yellow product changes into a complex known as a reduced form of phosphomolybdic heteropoly acid, which features an intense blue color.

*Dissolve (6.0 ± 0.1) g of ammonium molybdate in 200 cm^3^ of distilled water to prepare Reagent A*. Similarly, dissolve (0.15 ± 0.01) g of potassium antimony tartrate in 100 cm^3^ of distilled water. Both solutions should be gently heated until fully dissolved, then combined. Once the solutions are combined, pour the cooled mixture into 500 cm^3^ of a 5 mol/dm^3^ sulfuric acid solution and mix thoroughly. Next, adjust the total volume in a measuring flask to 1 dm^3^ with distilled water and remix. Store the solution in a dark, tightly sealed container in a light-protected area for up to 1 month. Refer to GOST R 54650–2011, Section 8.2.2, for further details.

*To prepare Reagent B*, dissolve (1.00 ± 0.01) g of ascorbic acid as outlined in section 6.18 in 170 cm^3^ of Reagent A. Then, transfer the solution to a 1 dm^3^ measuring flask and fill it to the mark with distilled water, ensuring thorough mixing. This solution should be prepared on the day of analysis to color both soil extracts and calibration solutions.

#### 2.7.1 Preparation of extracts from samples of mineral soil horizons

The soil samples used in the analysis weigh (10.0 ± 0.1) g and are placed in processing containers. An extracting solution of 50 cm^3^ is added to each sample. According to GOST R 54650–2011, the temperature of the extracting solution should be maintained within the range of (18 ± 3) °C, as monitored by a thermometer during use. Once the solution is added, the soil is mixed with the extracting solution in a mixer for 1 min before being allowed to settle for 15 min. After settling, the cassettes containing the suspensions are shaken manually and then filtered through paper filters. For further processing, the soil and extracting solution are mixed on a shaker for 15 min. After this, the suspensions are filtered through paper filters. To determine phosphorus compounds (P_2_O_5_), collect 2 cm^3^ of calibration solutions, filtrates of extracts, and 38 cm^3^ of reagent B in process containers. Measure the color of the solutions photometrically, no sooner than 10 min after adding reagent B.

#### 2.7.2 Calculation of the content of mobile phosphorus compounds (in terms of P_2_O_5_) in the soil

To calculate the amount of phosphorus compounds (expressed as P_2_O_5_) in the soil, use the concentration of mobile phosphorus compounds found in the soil extract along with the appropriate formulas. C_PM_ = 5xC, where: C_PM_ denotes the concentration of mobile phosphorus compounds, measured in terms of P_2_O_5_, within the soil extract. The coefficients 5 and 50 represent the ratio of the volume of the extracting solution to the mass of the soil, specifically for mineral and organic horizons, respectively. C signifies the concentration of mobile phosphorus compounds, also expressed in terms of P_2_O_5_, in the soil. According to the instructions and protocol for preparing the kit, we utilize the following table to determine the ratios of phosphorus compounds in the experimental boxes (Table 2).

**Table 2:** The ratio of concentrations of mobile phosphorus compounds (expressed as P_2_O_5_) in the soil extract and the soil (for mineral and organic horizons).

Experimental soil horizons	The concentrations ratio of mobile inorganic phosphorus				
The concentration of mobile phosphorus compounds (calculated as P_2_O_5_) in the soil extract	5	10	20	30	50
The concentration of mobile phosphorus compounds (calculated as P_2_O_5_) in soil (for mineral horizons), mg/kg soil	25	50	100	150	250

### 2.8 Data collection and statistical analysis

Statistical analyses were performed using Statistics for Windows version 6.0 and Microsoft Excel (2013 and 2016). Means and standard deviations (n–1) for three replicates were calculated with MS Excel’s data analysis tools. Mean comparisons were made using least significant difference (LSD) tests at a significance level of *p* ≤  0.05 with MSTAT-C software (version 6.1, Michigan State University, East Lansing, MI, United States).

## 3 Results

### 3.1 The effects of different doses of glyphosate on the number of bacteria in 1 mL of liquid medium at pH levels of 7.0, 8.0, 9.0, and 10.0 at 28 °C

**Figure 2:**
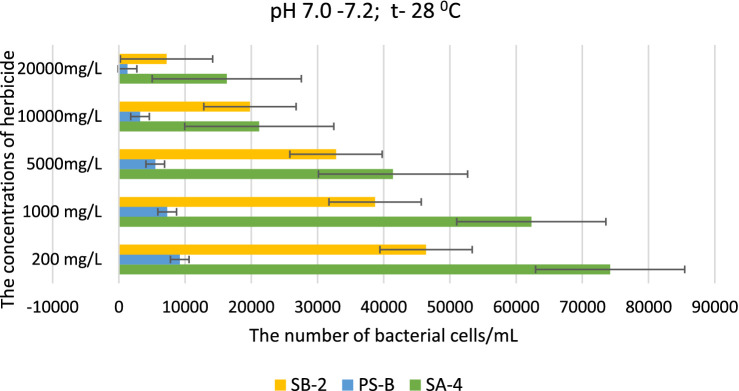
Effect of different doses of glyphosate on bacterial cell density in liquid medium at pH 7.0–7.2. Spectrometer analyses measured bacterial density at 550 nm; Values are given as mean ± SD, *n* = 3, and are significantly different at *p* ≤ 0.05.

Our research has revealed significant findings regarding bacterial resistance to herbicides, highlighting the need for further investigation. Strain SA-4, in particular, demonstrated a remarkable level of resistance compared to other bacterial species. Even when exposed to the highest dosage of 20.000 mg/L, the cells of this bacterium remained viable, albeit in insignificant numbers. For instance, a high number of SA-4 strain cells was recorded at 200 mg/L, reaching 74,200 ± 0.91 cells/ml. However, at a dose of 20,000 mg/L, only 16.300 ± 0.90 cells/mL were detected (Figure 2). In contrast, strain SB-2 exhibited moderate resistance to high concentrations of the herbicide, with the number of cells being approximately 1.5 times lower than that of strain SA-4. Strain Ps-B was found to be the most sensitive to the herbicide, exhibiting effects at a dose of 200 mg/L and accumulating comparatively low biomass, in contrast to the two tested strains. At 200 mg/L, its population was 2.3 times lower, amounting to only 9.200 ± 0.15 cells/mL. The growth patterns observed in Petri dishes highlight the differing sensitivities of various strains to high doses of glyphosate, which is a primary focus of our research. For example, strain Ps-B demonstrated a high sensitivity to the herbicide, with its cells counted in minimal quantities. In contrast, strain SA-4 exhibited resistance, yielding significantly higher units than the other strains (Supplementary Figure 1). This finding emphasizes the necessity of understanding the varying sensitivities of different strains to elevated doses of the herbicide. We can gain insights into their effects on bacterial abundance by comparing bacterial growth across different herbicide concentrations. This comparison suggests that while bacterial numbers may remain high at low doses of glyphosate, they decrease significantly as concentrations increase.

**Figure 3:**
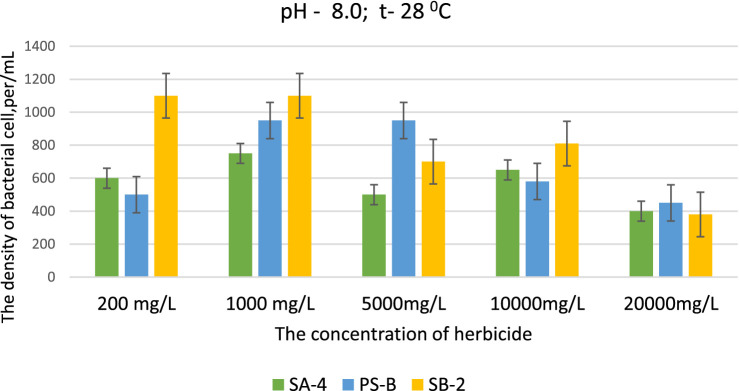
Effect of different doses of glyphosate on bacterial cell density in liquid medium at pH 8.0. Spectrometer analyses measured bacterial density at 550 nm; Values are given as mean ± SD, *n* = 3, and are significantly different at *p* ≤ 0.05.

As stated above, the experiments were designed to test a liquid nutrient medium with varying pH levels and increasing doses of glyphosate. The results were analyzed using spectrophotometry at 550 nanometers, a repeated process to ensure accuracy (*n* = 3). The primary goal was to study the effects of glyphosate on degraded soils with alkaline pH levels (above pH 8) at a temperature of 28 °C. As the data in Figure 3 show, the three strains studied showed different cell concentrations at pH 8.0 under the influence of different doses of herbicide. For example, strain SA-4 showed almost uniform growth at all doses of the herbicide, with the number of cells ranging from 663 ± 0.91 at 200 mg/L to 651 ± 0.91 cells/mL at 10000 mg/L of herbicide concentration. A noticeable decrease in the number of cells occurred only at a herbicide dose of 20,000 mg/L, but complete inhibition of growth did not occur. In contrast to other strains, Strain SB-2 demonstrated a more stable position, accumulating more biomass at nearly all doses, suggesting increased viability in an alkaline environment with elevated herbicide concentrations. For instance, this strain grew at herbicide concentrations of 200 mg/L and 1,000 mg/L, reaching 1,102 and 1,088 ± 0.91 cells/mL, respectively. This suggests that in a slightly alkaline environment (pH 8.0), the cells of this bacterium effectively absorb these herbicide doses. At higher concentrations of 5,000 and 10,000 mg/L, the bacterium also exhibited vigorous growth, resulting in a significant increase in cell numbers. A noticeable decrease in cell count was only observed at a herbicide dose of 20,000 mg/L, although complete growth inhibition did not occur at this level. In summary, the bacterium cells absorb herbicides well at pH 8, and growth inhibition is evident at 20000 mg/L herbicide exposure.

At a pH of 8, Strain Ps-B exhibited uneven growth compared to the other two strains when exposed to varying doses of glyphosate. Interestingly, this strain increased its cell density at a high dose of 5,000 mg/L, reaching 911 ± 0.91 cells/mL. As the concentration of the herbicide continued to rise, the cell density for this strain gradually declined. However, even at the highest dose, the cells continued to divide and produced new cells.

**Figure 4:**
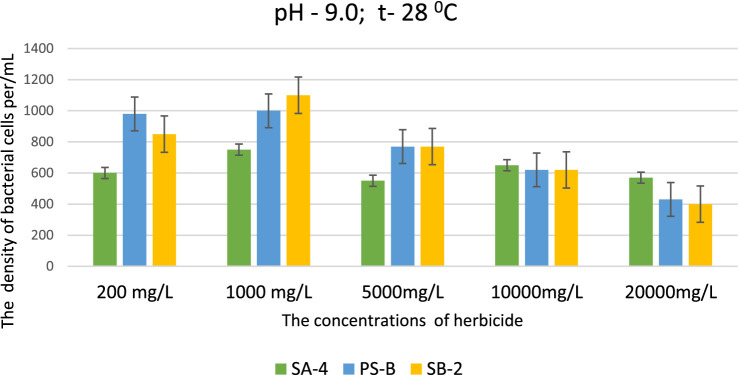
Effect of different doses of glyphosate on bacterial cell density in liquid medium at pH 9.0. Spectrometer analyses measured bacterial density at 550 nm; Values are given as mean ± SD, *n* = 3, and are significantly different at *p* ≤ 0.05.

**Figure 5:**
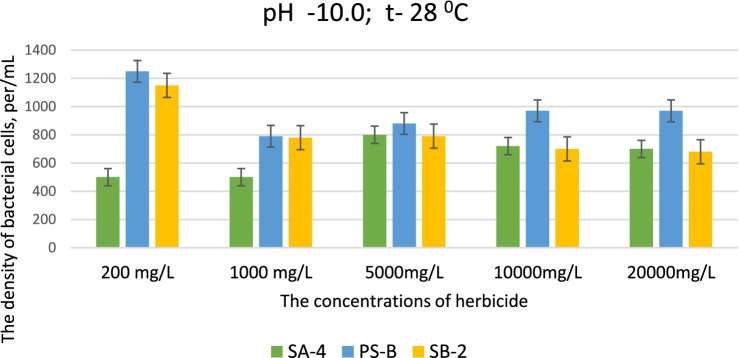
Effect of different doses of glyphosate on bacterial cell density in liquid medium at pH 10.0. Spectrometer analyses measured bacterial density at 550 nm; Values are given as mean ± SD, *n* = 3, and are significantly different at *p* ≤ 0.05.

This data is significant because the selected strains can effectively decompose high concentrations of herbicides, even in severely degraded soils. This ability is particularly remarkable in environments that are unfavorable for soil microorganisms and when soil processes are disrupted due to imbalances in pH levels, especially when they shift toward alkalinity. At pH levels of 9.0 and 10.0, strains SB-2 and Ps-B demonstrated active growth at glyphosate concentrations of 200, 1,000, 5,000, and 10,000 mg/L. Their cell density was significantly higher than that of strain SA-4, emphasizing their superior growth under these specific conditions. Even at a dose of 20,000 mg/L, especially Ps-B, had significant cell growth. All three strains can generally exhibit their degradative activity against this herbicide at high alkaline conditions (Figures 4, 5).

### 3.2 Determination of the minimum inhibitory concentration of glyphosate on the tested bacteria species in a solid medium

**Figure 6:**
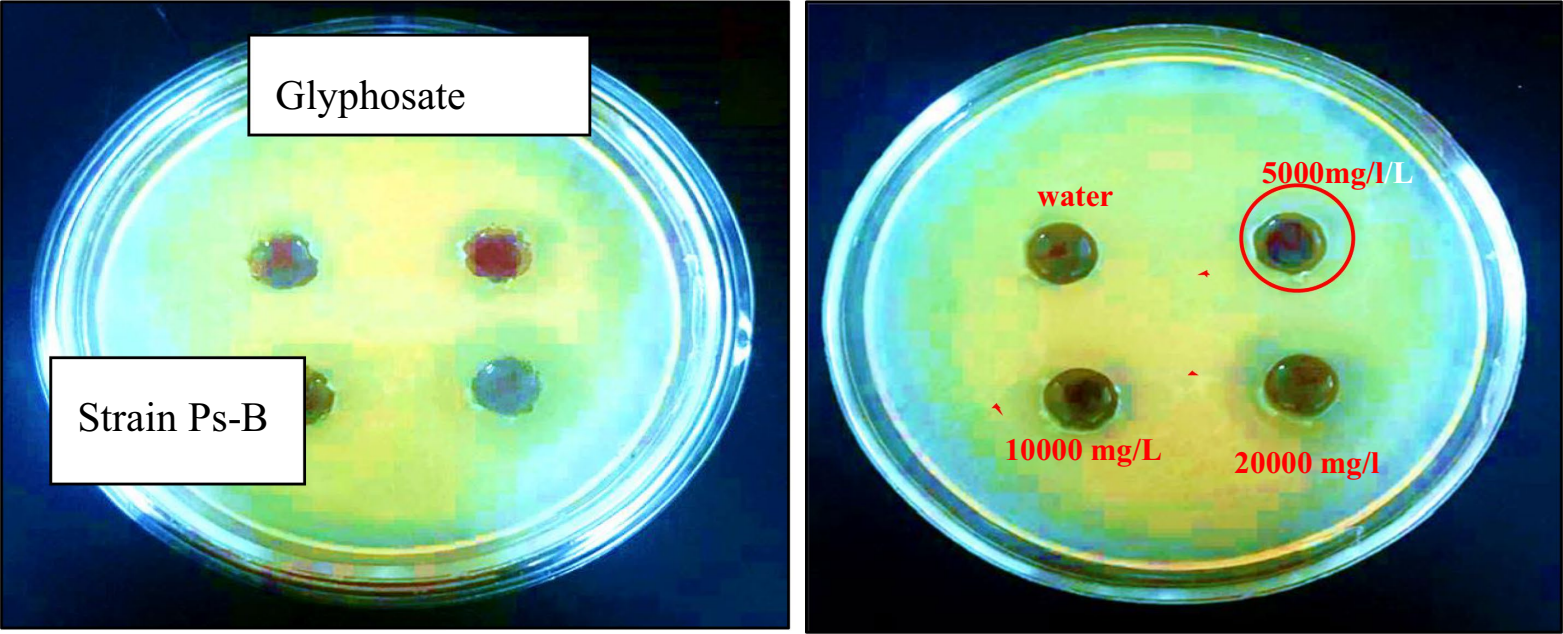
The minimum inhibitory concentration of glyphosate against *Stenotrophomonas sp*. Ps-B strain.

**Figure 7:**
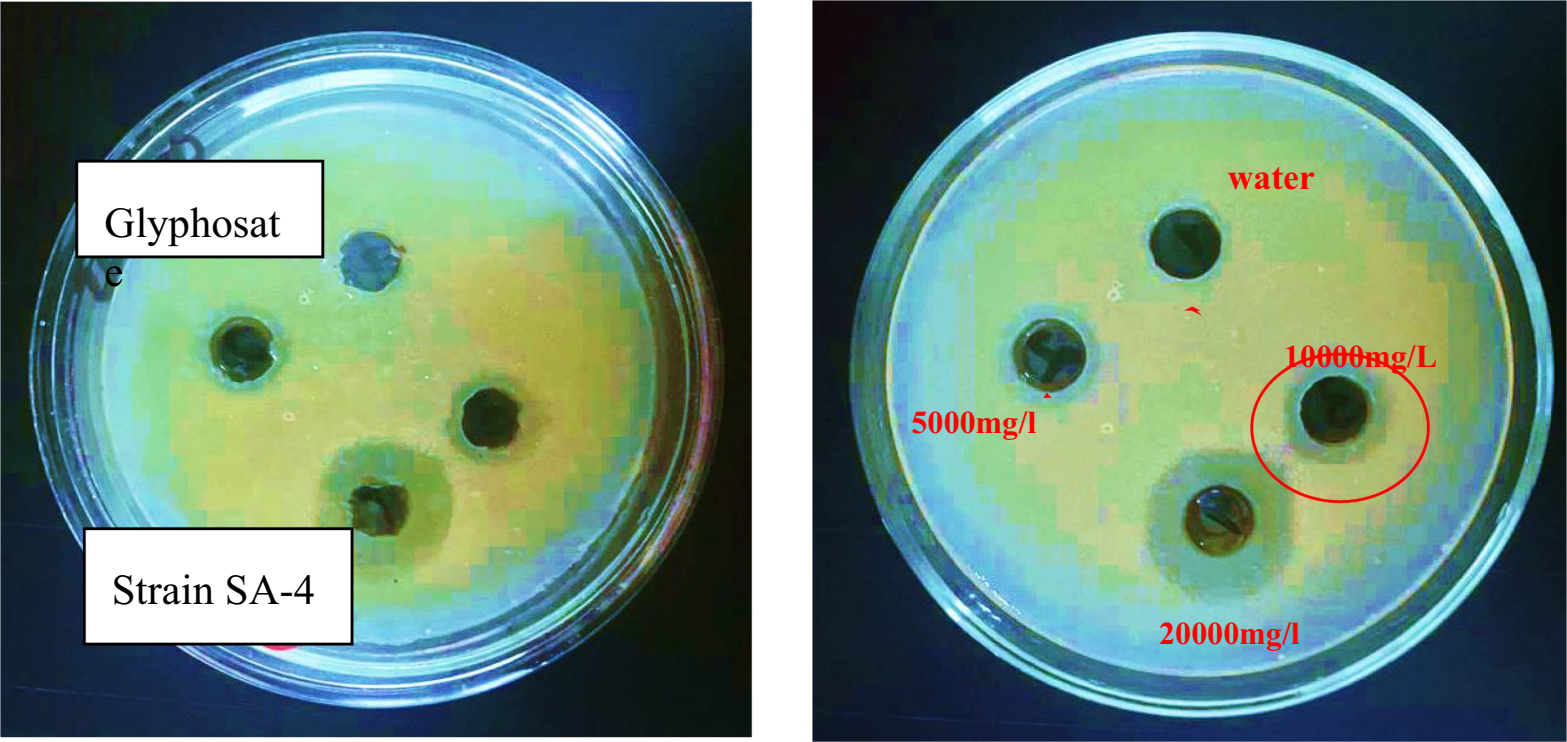
The minimum inhibitory concentration of glyphosate against *Lysinibacillus fusiformis*, SA-4 strain.

**Figure 8:**
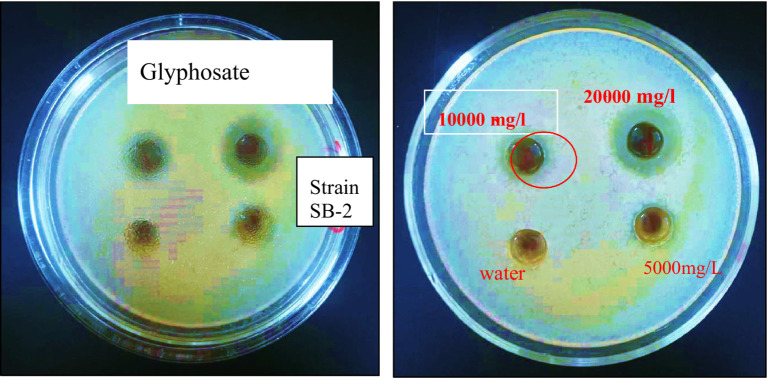
Minimum inhibitory concentration of glyphosate against the *Enterobacter cloacae*, SB-2.

The minimum inhibitory dose of herbicide on the viability of degrading bacteria in solid nutrient media was determined using the diffusion agar technique. The three bacterial strains exhibited varying levels of sensitivity to the herbicide glyphosate. The tests revealed that even a low dose of the herbicide effectively inhibited the growth of each of the three bacterial strains were examined at pH 7 and temperature 28 °C. According to the triple testing results, the minimum inhibitory concentration (MIC) of glyphosate for the Ps-B strain was determined to be 5,000 mg/L (Figure 6). Therefore, when considering the use of this strain in bioremediation, it is crucial to take into account the concentration of glyphosate present in the soil. If the glyphosate concentration in the soil does not exceed 5,000 mg/L, this strain can be employed to assist in removing the herbicide in neutral pH and medium temperature conditions. The minimum inhibitory concentration (MIC) of glyphosate for strain SA-4 was 10,000 mg/L (Figure 7). A concentration of 20,000 mg/L of glyphosate in the medium produced the largest zone of inhibition for this strain, measuring up to 12 mm. Strain SA-4 exhibits greater resistance compared to the Ps-B strain and can function effectively at higher herbicide concentrations in the soil. Unlike the Ps-B strain, SA-4 can initiate its degrading capability at doses of 10,000 mg/L or even higher in neutral pH and medium temperature conditions. If the soil contains glyphosate at concentrations of 10,000 mg/L, this strain can remove even larger amounts of the herbicide. The minimum inhibitory concentration of glyphosate for strain SB-2 was 10,000 mg/L (Figure 8). The largest zone of inhibition for the strain was approximately 8 mm, observed at a concentration of 20,000 mg/L. This strain can also degrade the herbicide at a concentration of 10,000 mg/L in the soil with neutral pH and medium temperature conditions.

### 3.3 Duration of glyphosate breakdown in soil with added bacterial species

**Figure 9:**
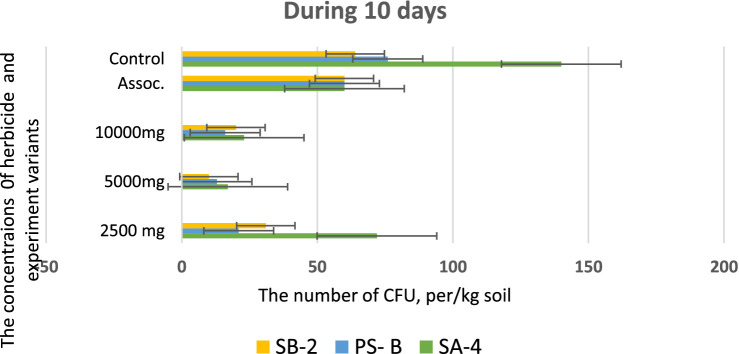
Bacterial counts at different glyphosate concentrations after 10 days of observation. Values are given as mean ± SD, *n* = 3, and are significantly different at *p* ≤ 0.05.

Following the initial screening, the degradation ability of glyphosate was tested in the laboratory on sterile soil that had not previously been exposed to the herbicide. The soil was disinfected by autoclaving for three consecutive days at 121 °C for 20 min. Different concentrations of glyphosate were mixed with the soil by adding sterile water and 10 mL of bacterial suspension (1х10^8^ cells/ml). Variants without added bacteria and without glyphosate served as controls. Soil samples were collected every 10 days and inoculated on nutrient media using the serial dilution method. The ability of three isolates to break down glyphosate at concentrations of 2.500 mg/kg, 5,000 mg/kg and 10,000 mg/kg was assessed. Intermediate products of glyphosate decomposed in the soil by isolates were assessed after 40 days. The final results of the studies were validated by sowing wheat seeds in these soils after 40 days. As shown in Figure 9, soils mixed with varying doses of glyphosate and incubated with bacterial cultures produced the following results after 10 days. At a high dose of 5,000 mg/kg, all strains exhibited similar outcomes, with the number of colony-forming units (CFUs) ranging from 25.0 ± 0.01 to 35.0 ± 0.01 per kg of soil. In contrast, at a lower dose of 2,500 mg/kg of glyphosate, the SA-4 strain demonstrated significantly better performance, achieving 75.0 ± 0.01 CFUs per kg of soil. Strain SB-2 also exhibited active reproduction at this concentration, yielding 36.0 ± 0.01 CFUs per kg of soil.

**Figure 10:**
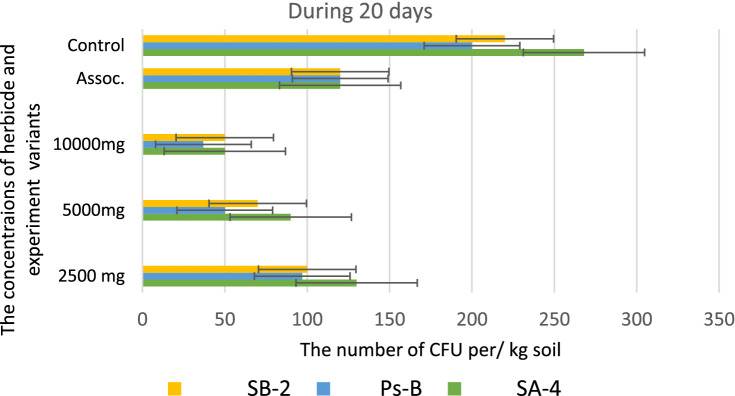
Bacterial counts at different glyphosate concentrations after 20 days of observation. Values are given as mean ± SD, *n* = 3, and are significantly different at *p* ≤ 0.05.

**Figure 11:**
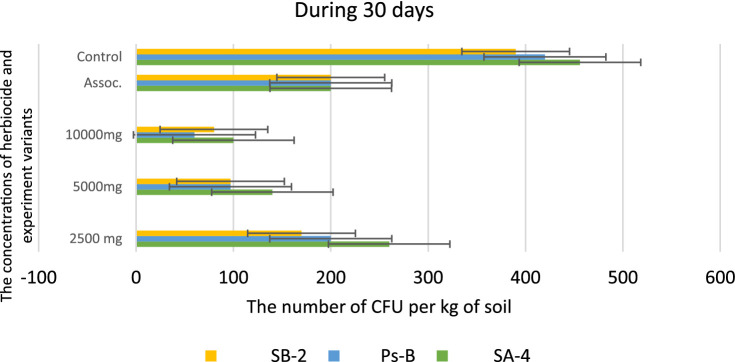
Bacterial counts at different glyphosate concentrations after 30 days of observation. Values are given as mean ± SD, *n* = 3, and are significantly different at *p* ≤ 0.05.

However, at the highest dosage of glyphosate (10,000 mg/kg), all strains showed decreased activity in colony formation, with CFUs counted as single units ranging from 12.0 to 15.0 ± 0.01 per kg of soil. In the trial where a combination of three bacterial strains was introduced to the soil containing the herbicide, strain SA-4 produced the highest number of colony-forming units, reaching 80.0 ± 0.01 per kg of soil. After 20 days in the soil with different concentrations of glyphosate, the introduced bacteria continued to multiply, especially at low doses of the herbicide. In the PS-B strain, the number of bacterial cells reached more than 1 × 10^2^ ± 0.01 CFU per g of soil, while the other two strains formed nearly 1 × 10^2^ ± 0.01 CFU per/g of soil. The cells of the PS-B strain demonstrated resistance even at a concentration of 5,000 mg, forming up to 1 × 10^2^ ± 0.01 CFU per g of soil. At a herbicide concentration of 1,000 mg, all strains produced a lower number in the soil, not exceeding 80.0 ± 0.01 cells. Nevertheless, in the association variant, the PS-B strain cells were able to form up to 1.5 × 10^2^ ± 0.01 CFU per g of soil. In contrast, the number of bacterial cells in the control soil was nearly twice that of the experimental bacterial cells (Figure 10). After 30 days of incubation in soil with degrading bacteria at various concentrations of glyphosate, an intensive reproduction of these bacteria was observed. The number of colony-forming units (CFU) in strain SA-4 reached 280.0 ± 0.01 at a dose of 2,500 mg/kg, while the other two strains at this dose managed to form CFU up to 180–200.0 ± 0.01 per g of soil. Additionally, the associations of these bacteria yielded more than 280 ± 0.01 CFU. In contrast, at doses of 5,000 and 10,000 mg/kg, the bacteria formed fewer than or slightly above 100.0 ± 0.01 CFU, demonstrating the toxic effect of glyphosate. However, compared to the measurements taken after 10 days, the number of colony-forming units at various glyphosate doses increased significantly after 30 days. This suggests that the process of herbicide utilizing in the soil continues, as the bacteria using glyphosate as a food source, leading to a substantial increase in their population. It appears that during this period, the biodegradation process either transformed or eliminated specific components present in the original samples, in contrast to the products obtained at the end of the experiment (Figure 11).

Moneke et al. (2010) demonstrated that bacterial genera endemic to soils and commonly found in permeable lining systems, such as *Bacillus, Pseudomonas, Azotobacter, Acetobacter,* and *Alcaligenes*, can thrive on mineral media using glyphosate as the sole carbon source. Our study confirms that when the glyphosate concentration reached 10,000 mg/L, the tested strains, both individually and in associations, survived by utilizing glyphosate as carbon and phosphate sources. Therefore, it can be concluded that the laboratory strains SA-4, PsB, and SB-2 can tolerate more than 10,000 mg/L of glyphosate. This is also consistent with the findings of Ezaka et al. (2018), who reported the transformation of glyphosate components during its degradation at the end of their experiment. The transformation may be due to microbial action or the action of plant enzymes. The use of plant growth-promoting bacteria will improve biodegradation and also restore the soil and biotic components (Wijekoon and Yapa, 2018; Esikova et al., 2023). Supplementary Figures 2, 3 show that the colonies of bacteria grown on the agar medium exhibit visual differences in density after 10 and 30 days. This confirms that, after 30 days, there was an increase in the number of colonies per gram of soil.

### 3.4 Determining the degradation intermediates of glyphosate in soil during incubation with bacteria that break it down

The pathways for glyphosate biodegradation vary depending on the type of bacteria and the enzymes they possess. For instance, in the bacterium *Pseudomonas* sp., the carbon-phosphorus lyase (C-P lyase) pathway is involved, leading to the degradation of glyphosate through the release of sarcosine and phosphate (Pipke et al., 1987; Masotti et al., 2021). The glyphosate oxidoreductase (GOX) pathway has also been identified in the bacteria *Arthrobacter* sp. GLP-1, *Flavobacterium* sp. GD1, *Ochrobactrum anthropic* and *Achromobacter* sp. resulting in the degradation of AMPA to methylamine and phosphate (Sviridov et al., 2012,^2015^; Ermakova et al., 2017).

Studies have revealed that the bacterial strains *Ensifer* sp. CNII15*, Acidovorax* sp. CNI26, *Agrobacterium tumefaciens* CNI28, *Novosphingobium* sp. CNI35, and *Ochrobactrum pituitosum* CNI52 utilize both C-P lyase and/or glyphosate oxidase degradation pathways to completely degrade AMPA (Rossi et al., 2021).

Several bacterial strains can utilize AMPK as a phosphorus source, but they are not capable of degrading the herbicide glyphosate itself. This suggests that the enzymes responsible for converting glyphosate into AMPK evolved independently of those involved in phosphorus metabolism (Acosta-Cortés et al., 2019). The sarcosine pathway uses glyphosate as a source of inorganic phosphate (Pi) (Dick and Quinn, 1995; Liu et al., 1991). However, the breaking of the carbon-phosphorus (C–P) bond in glyphosate relies heavily on the levels of both external and internal Pi. Consequently, this breaking generally occurs under phosphorus-deficient conditions, which are relatively uncommon in natural environments (Kertesz et al., 1991; Bazot and Lebeau, 2008). The breakdown of glyphosate involves breaking the carbon-phosphorus (C–P) bond, producing inorganic phosphate, which is a mobile and easily accessible form of phosphorus for plants. As a result, the microbial breakdown of glyphosate can increase the amount of mobile phosphorus in the soil. However, the success of this process depends on the availability of accessible phosphorus sources, as many bacteria are unable to use glyphosate as their sole phosphorus source (Zhan et al., 2018).

**Figure 12:**
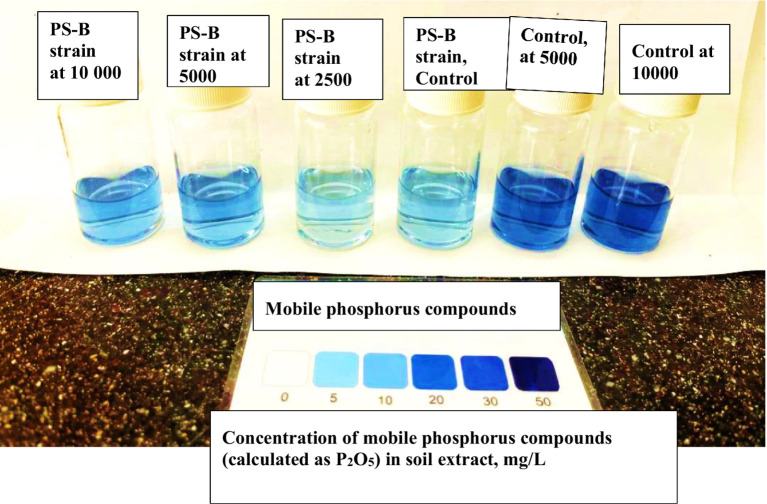
The color change on the scale shows the concentrations of mobile inorganic phosphorus compounds resulting from glyphosate decomposition, measured after 40 days of incubation in the soil using the PS-B bacterial strain.

**Figure 13:**
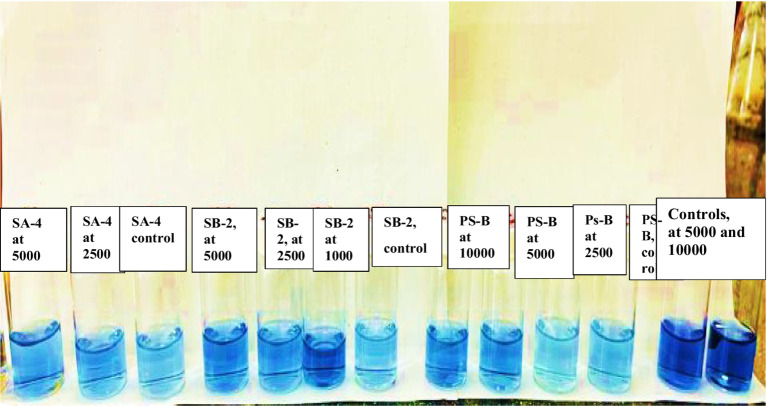
The color change on the scale shows the concentrations of mobile inorganic phosphorus compounds resulting from glyphosate decomposition, measured after 40 days of incubation in the soil using all tested bacteria.

To evaluate the decomposition of glyphosate after 40 days, the levels of mobile phosphorus compounds in the soil were analyzed. Following the specified method, the results were compared using color scales that indicate the concentration of mobile phosphorus compounds. As shown in Figure 12, a soil sample from the PS-B strains treated with a glyphosate dose of 2,500 mg/kg recorded a result of 5. Notably, as the glyphosate concentration increased, the levels of mobile phosphorus compounds also rose, moving from 5 to 20 on the scale. This trend was observed across all the strains studied. Interestingly, soil samples with only glyphosate showed a higher score of 30 on the scale for mobile phosphorus compounds. Additionally, in control soils treated with the studied bacteria only, without the herbicide, no values were recorded on the zero scale (Figure 13). The observed color change from pale blue to deep blue occurred in all studied strains, with the intensity increasing in correlation to the herbicide concentration. In the control samples, which contained only cultures of degrading bacteria in the soil and no herbicide, the color scale registered a value of 5, indicating the absence of the expected compounds. This absence—a key feature of our carefully designed control group—highlights the reliability of our research. In the control soils, where various concentrations of glyphosate were introduced without degrading bacteria, the color saturation scale showed values exceeding 30 and 50. This increase is likely due to the high concentrations of the herbicide in the soil, as the herbicide’s chemical compounds can react with the extraction solution, resulting in dark coloration.

Table 3 presents the calculated concentrations of mobile phosphorus compounds in soil experiments. These values were determined using the formula CPM = 5 × C, based on the specific data provided in Table 2. These results demonstrate the relationship between herbicide concentration, bacterial presence, and the observed saturation and color changes in the experimental variants. In the first control group containing only the herbicide (without added bacteria), a high herbicide concentration (1,600 ± 0.02 mg/L) was measured in the soil extracts. Conversely, in the second control group with only introduced bacteria (and no herbicide), the compound concentration was significantly lower at 400 ± 0.02 mg/L. This lower value reflects the baseline phosphorus compound content in the soil prior to glyphosate application. In soil samples treated with a glyphosate concentration of 2,500 mg/L, the activities of strains PsB and SB-2 remained at the control level of 400 ± 0.02 mg/L. This suggests that, for these strains, the herbicide dose was not sufficiently accessible for metabolic processes. As previously established, their activity is more pronounced under alkaline conditions (pH 9 and 10). In contrast, strain SA-4 demonstrated significantly higher activity in herbicide utilization, resulting in a calculated concentration of phosphorus compounds of 800 ± 0.02 mg/L. With increasing glyphosate concentrations, all strains showed increased yields of extracted phosphorus compounds, ranging from 800 ± 0.02 mg/L to 960 ± 0.02 mg/L. These findings are consistent with observed changes in color intensity, which correspond to higher yields of mobile phosphorus compounds.

**Table 3:** Estimated levels of mobile phosphorus compounds in soil experiments.

Concentration of mobile phosphorus compounds (calculated as P_2_O_5_) in soil extract, mg/L				
Control 2(only bacteria)	PsB	SB-2	SA-4	Control 1 (only glyphosate in soil)
	400 ± 0.02	400 ± 0.02	400 ± 0.02	–
at 2500 mg/L of Glyphosate	400 ± 0.02	400 ± 0.02	800 ± 0.02	1,600 ± 0.02 mg/L
at 5000 mg/L of Glyphosate	960 ± 0.02 mg/L	800 ± 0.02 mg/L	800 ± 0.02 mg/L	1,600 ± 0.02 mg/L
at 10000 mg/L of Glyphosate	960 ± 0.02 mg/L	960 ± 0.02 mg/L	800 ± 0.02 mg/Lл	1,600 ± 0.02 mg/L

### 3.5 Phytotoxicity test of soil after incubation with various glyphosate concentrations and degrading bacteria

Glyphosate, as an organophosphate compound, contains the following functional groups: PO3H2, –COOH, and –NH2 (Ren et al., 2014). Some studies have shown that the half-lives of glyphosate and its metabolites range from 10 days to 98 days (Bai and Ogbourne, 2016).

**Figure 14:**
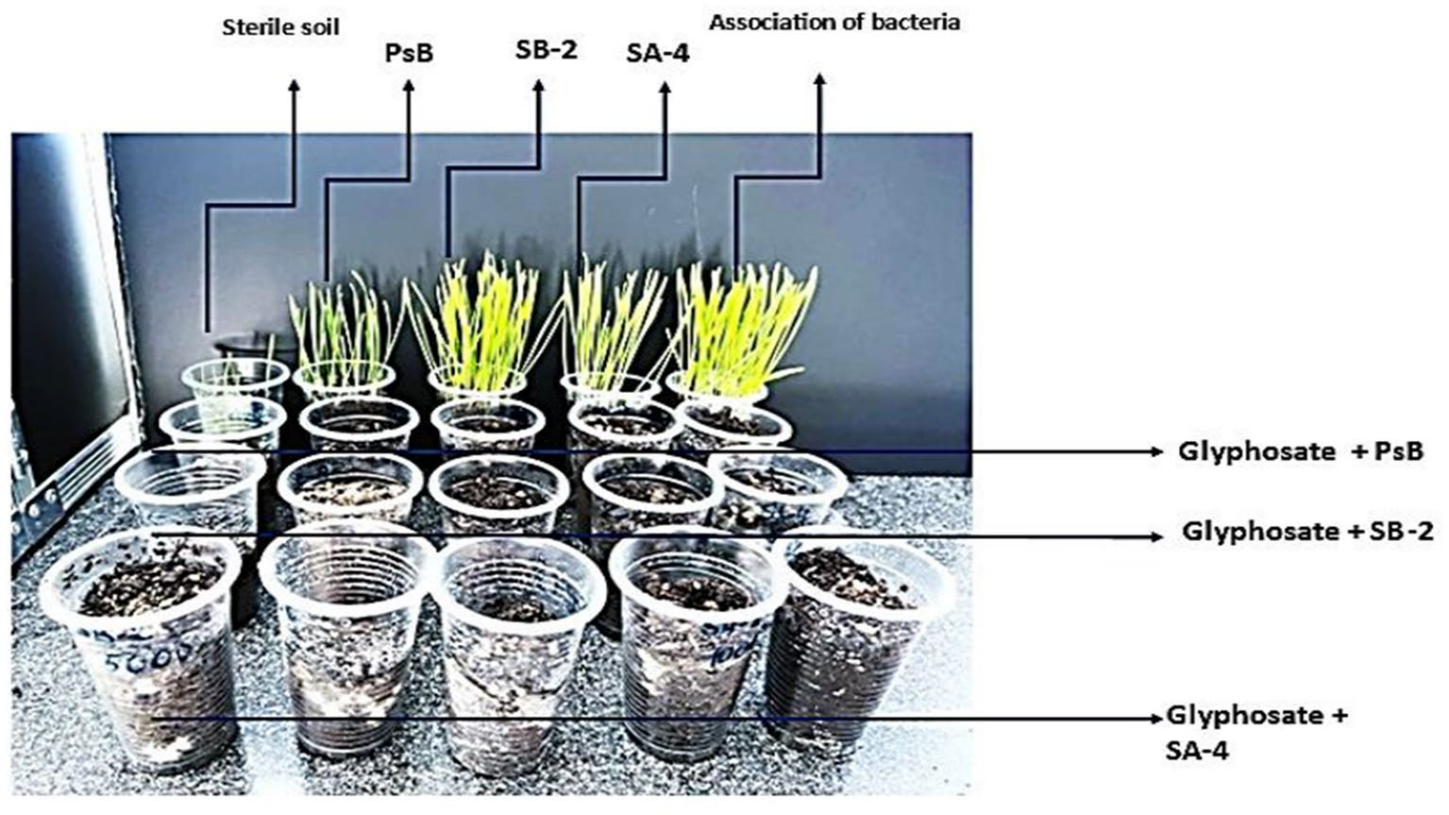
The impact of varying glyphosate concentrations and degradative bacteria on wheat seed germination and growth.

The literature reports that the half-life of glyphosate in soil can vary widely, ranging from 2 to 197 days depending on soil and climatic conditions. Under typical field conditions, the average half-life is estimated at 47 days (Weed Science Society of America, 2002; Tomlin, 2006). At the end of the study, after 40 days of incubation of bacteria with high glyphosate concentrations, the soil was inoculated with wheat seeds. As shown in Figure 14, seed germination and shoot growth were observed only in the variants that included bacteria alone. In contrast, there was no growth in the variants with a different glyphosate dose or in those that combined glyphosate with bacteria. This suggests that a period of 1 month plus 10 days may not be sufficient to complete the breakdown, and that high glyphosate concentrations have not yet decomposed into less toxic substances in the soil. However, the increase in the number of microorganisms suggests that active decomposition is occurring in the soil. To complete the decomposition of high doses of this herbicide, an additional incubation period of at least 40 days is required. However, the results of this study demonstrate that these bacterial isolates could be effective in cleaning soil after glyphosate application, helping to prevent its accumulation and reduce toxicity. Therefore, they could be utilized in the bioremediation of glyphosate-contaminated soil, even at high concentrations, for an extended period.

## 4 Discussion

Numerous studies have shown that biodegradation is a crucial and safe process for removing glyphosate from the environment by utilizing microorganisms, particularly bacteria. In our research, we carried out both *in vitro* and *in vivo* experiments to evaluate the ability of bacteria to use glyphosate as a source of inorganic phosphorus, nitrogen, and carbon, and to identify the concentrations at which it becomes toxic to these degrading bacteria.

Our results show that glyphosate transformation in soil is affected not only by bacterial activity but also by environmental physical properties. Soil characteristics and the applied glyphosate dose significantly influence its behavior. Both our experiments and previous studies reveal that in soils with high aluminum oxide and clay content, as well as low pH, glyphosate is strongly adsorbed and retained on the soil surface, leading to minimal immediate leaching (Cuhra et al., 2016; Soukup et al., 2020). These findings emphasize that physical soil factors and mechanical composition are key factors in herbicide degradation duration, and must be carefully considered when choosing soil type and pH for field bioremediation strategies (Zhan et al., 2018).

Developing effective bioremediation strategies for glyphosate requires a thorough understanding of how specific bacteria degrade this widely used herbicide. While glyphosate offers significant agricultural benefits, its persistence in the environment has raised considerable concerns and spurred research into microbial degradation methods. Recent studies have discovered bacterial isolates equipped with enzymatic pathways that break down glyphosate into less toxic compounds, underscoring the potential to reduce the environmental risks associated with its application (Pipke et al., 1987; Sviridov et al., 2012, 2015; Bazot and Lebeau, 2008). In this study, the selected bacterial isolates exhibited strong potential for glyphosate biodegradation, efficiently utilizing high concentrations of glyphosate across a range of pH levels. Through a three-stage screening process of the bacterial strains studied, we identified varying levels of glyphosate resistance, which were also influenced by environmental pH. Strain *Lysinibacillus fusiformis SA-4* demonstrated the highest resistance to elevated glyphosate concentrations at neutral pH. In contrast, the other two strains—*Stenotrophomonas* sp. *Ps-B* and *Enterobacter cloacae* SB-2 showed notable resistance at higher concentrations, but only under alkaline conditions (pH 9 and pH 10). These results suggest that bacteria from different taxonomic groups possess distinct metabolic capabilities and that the activity of enzymes responsible for glyphosate utilization as a nutrient source is strongly influenced by pH. Importantly, all tested strains tolerated substantial levels of the herbicide; even at concentrations as high as 20,000 mL/L, they survived, reproduced, and, at a minimum, maintained a detectable presence. So, the bacteria *Stenotrophomonas* sp. Ps-B, *Lysinibacillus fusiformis* SA-4, and *Enterobacter cloacae* SB-2 demonstrated significant potential at glyphosate concentrations several hundred times higher than the maximum allowable concentration (MAC). Our findings align with those reported by Castrejón-Godínez et al. (2021), who identified several key microorganisms involved in glyphosate degradation. Specifically, the studies highlighted bacteria from the genera *Achromobacter*, *Bacillus*, *Burkholderia*, and *Ochrobactrum*, as well as fungi from the genera *Aspergillus* and *Trichoderma*. Nonetheless, these authors emphasized that, without urgent and comprehensive research, there is a significant risk of underestimating the environmental accumulation of glyphosate and its metabolites. Currently, research on the practical application of microorganisms for glyphosate degradation remains limited. There is an urgent need for *in situ* studies aimed at harnessing microorganisms for effective glyphosate remediation.

The bacteria, through enzymes such as oxidases and C-P lyases, can cleave C-P bonds in the glyphosate intermediate, AMPA, and convert it to inorganic phosphorus (Jacob et al., 1988; Sviridov et al., 2012). Our study demonstrated that all three bacteria we examined could degrade the herbicide to inorganic phosphorus, indicating that they possess the same enzymes as previously studied microorganisms. Most prior studies focus on low doses and short degradation periods, typically ranging from 6 to 10 days.

Using a special method, we established precise inhibitory doses of the herbicide on the viability of degrading bacteria at pH 7 and a temperature of 28 °C. Among the strains tested, Ps-B was the least resistant, with a minimum inhibitory dose of 5,000 mg/L. In contrast, both SA-4 and SB-2 strains exhibited greater resistance, with a minimum inhibitory concentration of 10,000 mg/L for glyphosate. However, a review of the available literature reveals a lack of data on the ability of bacteria to degrade high concentrations, as observed in our experiments.

In some studies, the herbicide concentrations used did not go beyond 20 mg/mL. For instance, *P. aeruginosa*, *Bacillus cereus*, and a mixed culture degraded 7.2 mg/mL of glyphosate by 84.9, 72.7, and 66.4%, respectively (Fan et al., 2012). At a glyphosate concentration of 14.4 mg/mL, the degradation rates dropped to 47.15, 57.26, and 55.7%, respectively (Ezaka et al., 2018). Multiple studies (Zhao et al., 2015; Jacob et al., 1988) have reported that *Pseudomonas* sp. can degrade glyphosate, fully metabolizing up to 3.21 g/L. These studies also noted an efficiency of approximately 2 g of glyphosate degraded per gram of dry biomass.

Considering the abilities of the studied strains, we continued to evaluate their degrading activity in soil conditions at concentrations of 2,500 mg/kg, 5,000 mg/kg, and 10,000 mg/kg. We developed a simple and reliable method to monitor glyphosate degradation in soil using three bacterial strains, with the soil rendered sterile before their introduction. During a 40-day incubation at high glyphosate concentrations, the bacteria exhibited vigorous growth; their population increased steadily, suggesting they could use glyphosate as a nutrient source. The degradation process also resulted in the release of mobile phosphorus forms into the soil, indicating scales above 10 and 20. In contrast, when soils were treated with glyphosate but lacked the special bacteria to help break it down, they exhibited dark blue colors on the phytotoxicity scale, at values of 30 and 50. On this scale, higher numbers mean the soil is more toxic. This observation suggests that glyphosate undergoes biotransformation in soil. In particular, processes such as immobilization and leaching contribute to this transformation (Gómez Ortiz et al., 2017). Specifically, immobilization tends to occur rapidly and naturally, influenced by soil organic matter, mineral availability, clay content, and phosphorus concentration. Furthermore, the phosphoric acid structure in glyphosate binds to clay cations and organic matter in the soil, facilitating this process (Gómez Ortiz et al., 2017). Glyphosate can persist in soils with high levels of organic matter, phosphate, clay, aluminum, and iron, and it is easily leached under low pH conditions (Sidoli et al., 2016; Padilla and Selim, 2019). As a polyprotic acid, glyphosate binds to both anions and cations in soil over a pH range of 4 to 8 (Shushkova et al., 2009).

Our experiments revealed that in gray meadow soil with a pH of 7, higher amounts of glyphosate (2,500, 5,000, and 10,000 ppm) stayed in the soil for more than 40 days. This soil contains a relatively high amount of clay and aluminum, along with a significant amount of organic matter. Based on these characteristics and previous findings, we specifically hypothesize that, in the control variant using sterile soil without bacteria, the high concentrations of glyphosate added to the soil would become immobilized due to interactions with soil components. When extracted into the solution, phosphoric acid ions appeared dark blue, corresponding to a concentration scale of 50.

During this period, the soil remained phytotoxic, preventing wheat seeds from germinating. Even after introducing beneficial bacteria to assist the process, the phytotoxic effects persisted. These bacteria reproduced quite effectively, as shown by cell counts at 10, 20, and 30 days. For comparison, other studies have found that glyphosate can degrade over a period of 10 to 98 days (Bai and Ogbourne, 2016).

Based on these characteristics and previous findings, we specifically hypothesize that, in the control variant using sterile soil without bacteria, the high concentrations of glyphosate added to the soil would become immobilized due to interactions with soil components. When extracted into the solution, phosphoric acid ions appeared dark blue, corresponding to a concentration scale of 50. Our findings align with existing literature indicating that the mineralization of glyphosate into final or intermediate compounds is influenced by various factors. These include soil physicochemical properties, temperature, pH, and organic matter content (Zhang et al., 2015). Additionally, this mineralization process can vary over short periods under certain conditions (Nguyen et al., 2018). Several studies have shown that incubation temperature and organic amendments do not significantly influence the kinetic degradation curves of herbicides such as chlorotoluron and isoproturon in agricultural soils. The observed effects depended solely on the specific herbicide. However, the addition of organic amendments to soil decreased the degradation rates of both herbicides, likely due to increased sorption and reduced bioavailability for microbial degradation (Jesús et al., 2019). When organizing bioremediation of herbicides like glyphosate in field conditions, it is essential to consider these soil features.

## 5 Conclusion

Glyphosate has become widely used as a weed control agent in agricultural lands with meadow-gray and high-clay-content gray soils. Our research shows that it tends to persist and accumulate in these soils, increasing its harmful effects on natural ecosystems over time. This highlights the urgent need to improve biodegradation processes for this herbicide. We identified bacterial strains that tolerate high levels of glyphosate and convert it into a phosphorus source. These strains can be applied together, each employing unique mechanisms to reduce the herbicide’s toxic effects. The results identified the minimum threshold doses of glyphosate herbicide that inhibit bacterial degradation. Each degrading bacterium has a specific concentration above which it cannot metabolize the herbicide. Knowing these thresholds is crucial for effective bioremediation in contaminated soils. The threshold inhibitory concentration of glyphosate is 10,000 mg/L for *Lysinibacillus fusiformis* SA-4 and *Enterobacter cloacae* SB-2, and 5,000 mg/L for *Stenotrophomonas* sp. Ps-B. *Stenotrophomonas* sp. Ps-B shows higher degradative activity at pH levels of 8.0, 9.0, and 10.0, which is important for biodegradation in alkaline soils.

The rate at which glyphosate degrades is influenced by several factors, including the herbicide’s concentration, the mechanical and chemical properties of the soil, pH levels, and the activity of degrading bacteria. Given the risks associated with the widespread and annual application of glyphosate, which can accumulate in soil by binding to organic matter and clay particles, it is essential to explore safe and effective remediation methods. The strains *Lysinibacillus fusiformis* SA-4, *Enterobacter cloacae* SB-2, and *Stenotrophomonas* sp. Ps-B, effective at 5,000 mg/L and 10,000 mg/L, are recommended for bioremediation of soils heavily contaminated with high doses of glyphosate.

## Data Availability

The datasets presented in this study can be found in online repositories. The names of the repository/repositories and accession number(s) can be found at: All relevant data is contained within the article: The original contributions presented in the study are included in the article/Supplementary material, further inquiries can be directed to the corresponding author/s.
