# Supplementary material for: The bacterial species’ degradation activities at maximum threshold doses of glyphosate across different pH levels and temperature glyphosate biodegradation by soil bacteria at high doses under variable pH and temperature
Source: Front Microbiol. 2025 Dec 4;16:1668968. doi: 10.3389/fmicb.2025.1668968 (PMC12712341; doi:10.3389/fmicb.2025.1668968)
Supplement: Supplementary file 1 [file Data_Sheet_1.pdf]

frontiers

Supplementary Material

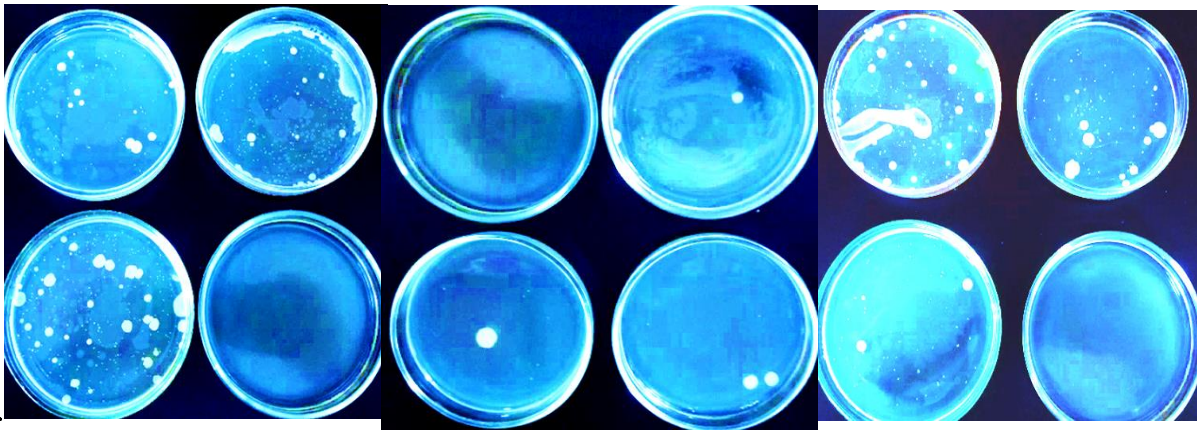

SA-4 **Ps-B** **SB-2**

Supplementary Figure 1. The growth rate of degrading bacteria colonies in the medium with different concentrations of glyphosate.

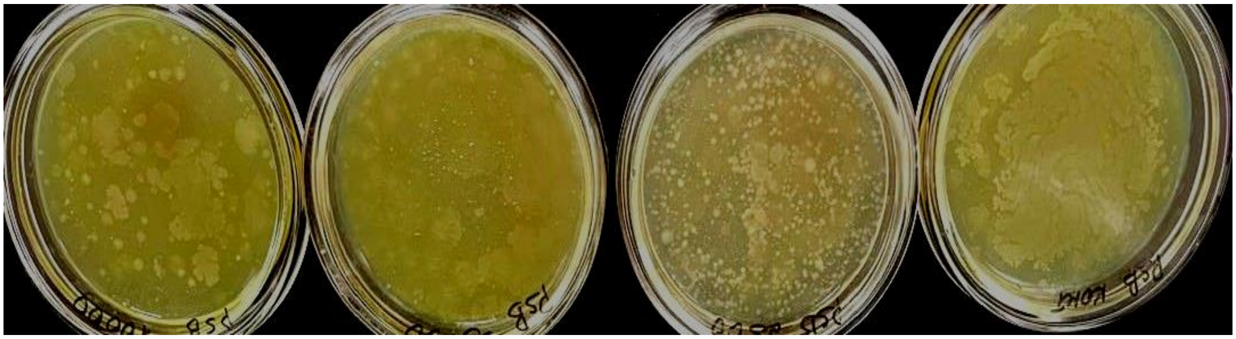

Supplementary Figure 2. Colonies of bacterial associations on agar medium after 10 days of incubation in soil with various concentrations of glyphosate.

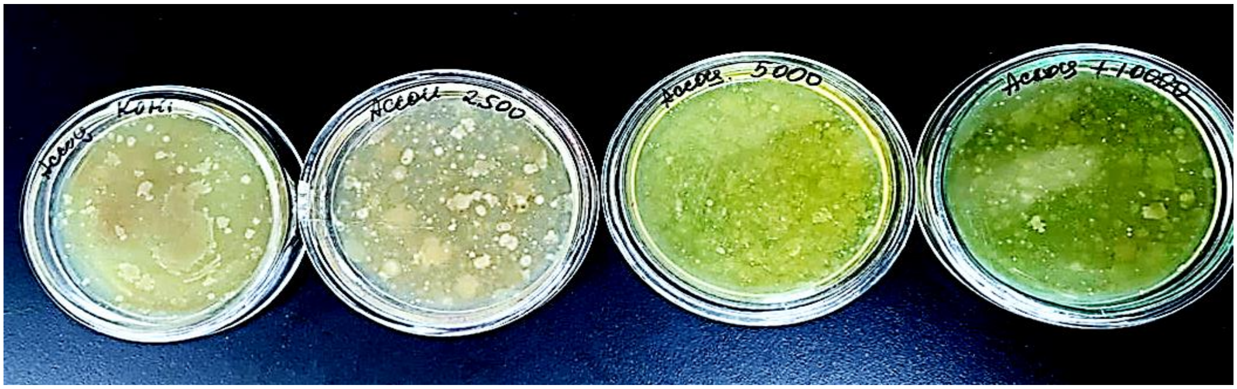

Supplementary Figure 3. Colonies of bacterial associations on agar medium after 30 days of incubation in soil with various concentrations of glyphosate.
